# THINGS LEFT UNDONE: RELATION OF GRIEF TO SENSE OF PURPOSE IN BEREAVED OLDER ADULTS

**DOI:** 10.1093/geroni/igac059.2135

**Published:** 2022-12-20

**Authors:** Shubam Sharma, Emily Mroz, Susan Bluck

**Affiliations:** Kennesaw State University, Kennesaw, Georgia, United States; Yale University, New Haven, Connecticut, United States; University of Florida, Gainesville, Florida, United States

## Abstract

Sense of purpose is grounded in life experiences (Ko et al., 2019) and can be threatened by the loss of a loved one. The death of a spouse in late life can be particularly disruptive when the bereaved struggle with perceptions of unfinished business with the deceased. This mentality may also derail their sense of life purpose. This study examined the association between persistent grief symptomatology (Prigerson et al.,1995) and sense of purpose (Ryff, 1989) in conjugally-bereaved older adults (N = 53; Mage = 81.59). Unfinished business constructs (i.e., unfulfilled wishes, unresolved conflict; Holland et al., 2018) were tested as mediating grief-purpose relations. Older adults who reported greater grief symptomology also experienced lower sense of purpose (r = -.27, p < 0.05). Extent of unfulfilled wishes fully mediated the relation between grief symptomology and sense of purpose (t = -2.26, p < 0.05); unresolved conflict did not act as a mediator. Findings indicate that the association between experiencing pervasive grief symptomology and a disrupted sense of purpose may be due, in part, to ongoing rumination about unfulfilled wishes: missed opportunities to meaningfully engage with one’s spouse before death. Results are poignant given recent COVID-19 physical distancing requirements that separated older couples during spouses’ dying days, potentially prompting unfulfilled wishes across bereavement. Findings highlight the need for palliative care practices that facilitate optimization of relationships at end of life and grief support practices that re-ignite purpose after difficult spousal loss.

